# Neurodegeneration Behind Bars: from Molecules to Jurisprudence

**DOI:** 10.3389/fpsyt.2014.00115

**Published:** 2014-08-27

**Authors:** Adonis Sfera, Carolina Osorio, Roberto Gradini, Amy Price

**Affiliations:** ^1^Department of Psychiatry, Patton State Hospital, Patton, CA, USA; ^2^Loma Linda University, Loma Linda, CA, USA; ^3^Department of Pathology, Sapienza University of Rome, Rome, Italy; ^4^Evidence Based Health Care, University of Oxford, Oxford, UK

**Keywords:** frontotemporal dementia, m’Naughten rule, von Economo neurons, saience network, neurodegenerative disorders

The prison population is aging and developing neurodegenerative disorders at a faster pace than the general population. Hidden among this group of recidivist, career criminals, there is a subpopulation of first offenders with frontotemporal dementia behavioral variant (bvFTD). The pathological hallmark of this condition is frontotemporal lobar degeneration (FTLD), which early on spares cognition, yet predisposes to criminal violations. From the neurobiological perspective, bvFTD originates in a large-scale brain network in charge of motivation and concern, the salience network (SN). From the judiciary perspective, bvFTD is challenging because patients often retain the “appreciation” of right and wrong, yet may be organically incapable to act accordingly. Equally challenging are the dispositions in regards to bvFTD patients: return to the community, risking further violations, vs. incarceration with dismal punitive or rehabilitative benefit. In this article, we advocate for screening of all first offenders who are 55 years of age or older via neuropsychological testing and/or positron emission tomography (PET) and should bvFTD be diagnosed, their placement in, yet to be developed, palliative programs in state or private facilities.

## Introduction

It is estimated 40,000 inmates with dementia are currently incarcerated in U.S. prisons with forecasted increases topping a quarter of a million persons by 2050 (1). The reasons for the graying of prison population are multiple, including longer incarceration periods, mandatory prison sentences, and an increase in the number of older first offenders (2). It is also believed that people in prison age at a faster rate than the general population (3). The category of older first offenders has increased steadily over the past two decades. According to the Human Rights Watch report, the number of persons over 60 years of age entering state prisons as new court commitments grew by 109% between 1995 and 2009 (4). Recent studies demonstrate that 54% of bvFTD patients commit criminal acts (5) and since this condition represents 5–6% of all dementias, it is possible that it accounts for the large numbers of older first offenders entering prisons. Moreover, up to 51% of bvFTD patients are misdiagnosed at the initial evaluation because the symptoms resemble psychiatric conditions and are missed by routine cognitive testing (6). In this opinion article, we emphasize a multidisciplinary approach to bvFTD; we believe that input from disciplines such as neurobiology, psychology, psychiatry, forensic psychiatry, and jurisprudence could help us comprehend better this complex disorder.

## Neurobiology of the Salience Network

### The network

It has been known for almost a century that frontotemporal lesions lead to anti-social acts (7). The reason for this condition is not entirely clear, but it has been hypothesized that the anterior insular cortex (AIC) and anterior cingulate cortex (ACC), especially in the right hemisphere, harbor pro-social cognition, such as the sense of fairness and playing by the rules and that lesions in these nodes result in criminal violations (8). Currently, this represents the working hypothesis attempting to explain why 54% of patients with bvFTD are involved in criminal behavior, compared to only 12% of patients with Alzheimer’s disease (5).

Recent studies demonstrated that AIC and ACC in the right hemisphere are in charge of implementing and adjusting mental models to social contexts, for example, concern for the rule of law and civilized behavior (9). Patients with bvFTD may understand legal and societal rules, however, they seem to present with dissociation between the knowledge of wrongfulness of acts and the concern for avoiding them.

The pathology in bvFTD consists of FTLD, which originates in the ACC and AIC, mainly in the right brain hemisphere. This focal lesion represents the starting point of a unique trail leading, via psychological and anthropological domains, to jurisprudence. Functional neuroimaging studies combined with brain network analysis place these nodes in the SN, which is one of the large-scale brain intrinsic connectivity networks (ICN) (10, 11). It is involved in selecting and attributing salience to external or internal stimuli. Three ICNs have implications for bvFTD: the central executive network (CEN), the SN, and the default mode network (DMN) (12) (Table 1).

**Table 1:** **The three major ICNs and their implications in bvFTD**.

ICN	Cortical location	Function	Social context
CEN	Dorsolateral prefrontal cortex (DLPFC), supramarginal gyrus	Planning, working memory, direction of attention	Outer reality, task, actions, problem-solving
DMN	Posterior cingulate cortex, medial prefrontal cortex, medial temporal lobe, and angular gyrus	Self-related mental activity, including autobiographical, self-monitoring, and social functions	Inner reality, thoughts, ideas, societal rules, self, and the community
SN	Anterior cingulate cortex (ACC) and frontoinsular cortex (FIC)	Detection and orientation to salient external stimuli and internal events	Switches the focus of concern back and forth from the inner to the outer reality

In addition to its cortical areas, SN comprises subcortical nodes, such as amygdala, ventral tegmental area, substantia nigra, and the thalamus (13). Together the cortical and subcortical components of the SN, engender a saliency-motivation-action (SMA) system, which translates selected thoughts and ideas into action. This process may function as a filter that prioritizes informational input by tagging it with various degrees of salience (14). Input raising enough concern and requiring immediate physical or mental action is prioritized and enacted.

Neuroimaging studies demonstrate AIC activation during risk evaluation, suggesting the insula may play a role in human concern about obstacles in reaching targets (15). The ACC activates during evaluation of prediction models or reward related activities, suggesting a connection between anticipation, motivation, and action (16). Direct stimulation of ACC triggers motivation and readiness to act: in case reports of direct electrical stimulation of the ACC, patients reported having a sensation of imminent challenge coupled with a determined attitude to overcome it (17). Ability to abide by the rule of law or the societal code of conduct requires mental operations such as risk evaluation, prediction of behavioral outcome, and motivation to avoid negative outcomes, capabilities, which patients with bvFTD do not possess.

### The cells

At the cellular level, the SN is home to von Economo neurons (VENs). VENs are large projection neurons with equally large axons and high conduction speed, qualities, which enable them to integrate multiple signals from distant brain areas (18, 19). Recent studies demonstrate that FTLD originates and preferentially targets VENs in the right ACC and AIC. Interestingly, there are 30% more VENs in the right hemisphere (20). Since empathy and social intelligence are impaired in bvFTD, it is believed that these qualities are lateralized in the SN of the right hemisphere, containing more VENs (21). The right hemisphere is known to participate in social intelligence, which includes the need for affiliation with peers, while the left contributes to individuation, competition, and differentiation from the group (22, 23). In bvFTD, the VENs population of ACC is reduced by an average of 53–74% and most surviving VENs are severely altered (18, 24). Interestingly, suicide completers have a higher number of VENs, suggesting that over-attribution of saliency may overwhelm this network (20). The role of VENs’ in salience processing is further suggested by the fact that these neurons express high levels of disruption in schizophrenia 1 (DISK 1) protein, known to be associated with the ability to shift attention (25).

### The molecules

At the molecular level of VENs, bvFTD was shown to target either the DNA-binding protein TDP-43 or the microtubules associated tau protein (MAPT) (26, 27). Tau protein is known to be a microtubule (MT) stabilizer, while TDP-43 forms cytoplasmic mRNP granules that are transported via MTs to distal neuronal compartments (28). MTs were demonstrated essential in information processing and memory encoding (29). For example, hyper-phosphorylation of tau, which is known to impair MT assembly and structure also impairs cognition (30). VENs are known by their large diameter axons, which may contain the highest proportion of MTs in the CNS. The loss of VENs in FTLD may be secondary to the loss of MTs, and thus constitute the reason that these neurons are preferentially targeted by this neurodegenerative process. In addition, biophysical evidence documents the ability of MTs to process information by accessing logic gates in transistor-like fashion, as well as by the ability to reorganize their molecular structures in response to electromagnetic fields (31–33). Taken together, these capabilities of MTs suggest a role in information and saliency processing.

### Psychology and anthropology

From a psychological perspective, the loss of MTs and VENs may manifest as impaired social cognition. Psychological traits engendered by the SN and lost in bvFTD include: detection of deception, empathy, detection of social error, motivation, risk prediction, recognition of error, awareness, insight, risky decisions, rapid intuition, detecting malevolent intentions in others, viewing faces of allies.

Anthropologically, the human civilization was ultimately engendered by the human brain. Over time, the SN may have sharpened human attention and concentration, enabling symbolic representation, which is the stepping stone of civilization (34, 35). By affecting the same brain networks that contributed to the development of human civilization, FTLD may regress the thought process to a pre-symbolic stage that dominated human thinking prior to the existence of the civil society and the rule of law. For example, this stage may have been depicted by Aeschylus (456 BC) in “Oresteia” trilogy, which chronicles the House of Atreus, disintegrating under a curse consisting of a primeval cycle of blood-revenge. This curse is reversed by the implementation of a civil society based on the rule of law (36).

## Jurisprudence and the Salience Network

Disturbed individuals who commit crimes remain dilemmas for society since ancient times. Plato, for example, believed that such individuals were not responsible for their acts the way others were and should not be punished by the same means (37).

The majority of patients with dementia do not engage in violent acts, however, about a third are aggressive, presenting with agitation, paranoia, wandering, and sexual disinhibition. These behaviors can bring them into conflict with the criminal justice system (38). In bvFTD assessing competency to stand trial (CST) and determining culpability and suitable sentencing is especially challenging because these defendants are sometimes able to appreciate the wrongfulness of their acts and might understand societal rules, but may be organically incapable of regulating their behavior (39). Currently, in the US, the M’Naughte rule (United States Code, Title 18, Section 17) defines legal incapacity of a defendant as being “unable to appreciate the nature and quality of the wrongfulness of his acts.” According to M’Naughten rule, patients with bvFTD would not qualify to plead “not guilty by reason of insanity” (5).

A similar situation is encountered in the assessment of the capacity to stand trial (CST). The legal standard for CST is “whether the individual has sufficient present ability to consult with his lawyer with a reasonable degree of rational understanding and whether he has a rational as well as factual understanding of the proceedings against him (40, 41).” Frequently psychiatric reviews leave these accused in legal limbo, waiting to regain competency in facilities embracing recovery models (42).

In the case of Alzheimer’s disease, studies demonstrate that 30–50% of criminal defendants older than 60 are incompetent to stand trial (IST) (43), but no similar studies have been completed in bvFTD.

We are in need of a modern system for placement of individuals with neurodegenerative disorders. Such a system, based on the palliative model, would benefit elderly offenders diagnosed with dementia, while at the same time help decongest correctional facilities not designed to accommodate patients with neurodegeneration.

We recommend task forces at both federal and state levels to study the issue of palliative placement of incarcerated dementia patients as well as screening first offenders 55 years of age or older for bvFTD. The screening should follow the criteria of the 2011 International Behavioral Variant Frontotemporal Dementia Criteria Consortium (BVFDC). These criteria consist of six clinical hallmarks, including: disinhibition, apathy/inertia, loss of sympathy/empathy, perseverative/compulsive behaviors, hyperorality, and dysexecutive neuropsychological profile. According to BVFDC, at least three of these features must be present for the diagnosis of “possible” bvFTD. “Probable” bvFTD requires the presence of functional disability and characteristic neuroimaging in addition to clinical symptoms. “Definite” diagnosis of bvFTD needs either documentation of FTLD by histopathological confirmation or a pathogenic mutation (44).

## Conclusion

The difficulty in distinguishing symptoms of bvFTD from antisocial personality disorder or psychosis constitutes the reason individuals with this condition can be found in correctional institutions. FTLD of the structures comprising the SN renders these defendants organically incapable of concern with social norms or the rule of law. This interesting observation offers a unique glimpse in the mechanisms the brain uses in selecting salient stimuli from the background noise of irrelevant input.

The criminal justice system and the correctional institutions were not designed to accommodate persons with dementia. Most forensic psychiatric programs are founded on the recovery model geared toward the mentally ill expected to regain CST and is not adequate for individuals with neurodegenerative disorders. Appropriate care of these patients requires the development of preventative programs for screening and placement in palliative care facilities.

## Conflict of Interest Statement

The authors declare that the research was conducted in the absence of any commercial or financial relationships that could be construed as a potential conflict of interest.
